# Transversus Abdominis Thickness at Rest and Exercise in Individuals with Poststroke Hemiparesis

**DOI:** 10.3390/sports8060086

**Published:** 2020-06-12

**Authors:** Anna Kelli, Eleftherios Kellis, Nikiforos Galanis, Konstantinos Dafkou, Chrysostomos Sahinis, Athanasios Ellinoudis

**Affiliations:** 1 Private Physiotherapy Clinic, Kavala 65403, Greece; physiokelli@gmail.com; 2 Laboratory of Neuromechanics, Department of Physical Education and Sport Sciences at Serres, Aristotle University of Thessaloniki, Serres 62100, Greece; kdafkos@phed-sr.auth.gr (K.D.); chrysosto@phed-sr.auth.gr (C.S.); ellinoud@phed-sr.auth.gr (A.E.); 3 School of Medicine, Aristotle University of Thessaloniki, Thessaloniki 54124, Greece; kyros@auth.gr

**Keywords:** ultrasonography, muscle size, stroke, hemiplegia, biomechanics

## Abstract

The activity of the transverse abdominal (TrA) muscle affects the stabilization of the trunk. It is known that after a stroke, people experience problems in performing daily activities. The purpose of this study was to examine whether there are differences in the transversus abdominal thickness between the two sides of the body in individuals with hemiparesis and controls. Eight patients with hemiparesis and nine controls matched for age and body mass index were examined by musculoskeletal ultrasound in four conditions: a) At rest, b) abdominal hollowing maneuver from the supine position, c) bridge, and d) abdominal hollowing maneuver from the bridge position. In each of the above conditions, the symmetry index was calculated as the absolute value of the difference in thickness between the two sides. Analysis of variance showed a lower TrA thickness at rest and exercise in patients compared to the control group (*p* < 0.05). Further, patients showed a lower contraction thickness ratio during exercise compared to controls (*p* < 0.05). The absolute symmetry of the TrA thickness was 12.59 ± 6.43% to 19.31 ± 10.43% in patients and it was significantly greater than the control group (3.01 ± 2.47% to 4.47 ± 2.87%). According to the above results, it seems that transverse abdominal activation exercises are particularly useful for improving the stability of patients with hemiparesis, as long as they are located and adapted to the deficit of each patient.

## 1. Introduction

Stroke is a clinical syndrome, which is characterized by a variety of signs of disturbance of the central nervous system function. Poststroke hemiparetic individuals often present motor impairments, such as difficulties in performing daily tasks [1,2]. These impairments are accompanied, amongst others, by muscle atrophy [3,4], a reduction of muscle strength [1,5], and changes in anticipatory muscle activation [6], which ultimately have a negative impact on the quality of life in these patients [7].

Poststroke patients frequently have problems associated with controlling the trunk [8,9]. This may be related to atrophy and altered activation of the trunk muscles [3,4,6]. Trunk stabilization muscles include the lumbar multifidus complex as well as the transversus abdominis (TrA) [10,11]. Individuals with pain and trunk instability often display a delay in activating the TrA [12,13,14,15], which improves significantly following therapeutic exercise interventions [16]. Hence, the quantification of atrophy and activation of TrA after stroke is worthwhile.

Ultrasonography represents a non-invasive technique, which is currently used to examine TrA thickness at rest and exercise, thus allowing examination of the TrA muscle size and function [17]. To our knowledge, only one study has examined TrA thickness in poststroke patients. In particular, Kim et al. [18] reported that the TrA thickness at rest of both sides of the body was lower in individuals with hemiparesis than controls. Similarly, the increase in thickness during the abdominal hollowing maneuver (ADIM) was also lower in patients than controls. This is in line with previous investigations on muscle atrophy [3,4] following stroke and indicates that ultrasonography can assist in better identifying atrophies of the deep abdominal muscles following stroke.

Poststroke patients with hemiparesis present impairments in the function of one side of the body relative to the other [6] due to the appearance of muscle weakness contralateral to the brain lesion [19]. Although stroke affects unilateral limb activity, it has the potential to deteriorate the function of the trunk muscles on both sides of the body, affecting the proximal control [8,9]. In fact, the muscle cross-sectional area of the contralateral (to the brain lesion) paravertebral muscles appeared to be larger than the ipsilateral side in individuals with unilateral hemiplegic stroke [9]. In addition, patients with stroke showed asymmetrical activation of the lateral trunk muscles because of a lower activation of the paretic body side [6]. Thus, therapeutic exercises that aim to improve trunk muscle strength, improve stability, and reduce bilateral asymmetries in stroke patients are an important element of exercise interventions. In healthy individuals, Rankin et al. [20] showed that the TrA thickness showed an asymmetry of, on average, 16% to 24%, depending on the scanning site. Very few studies have examined TrA thickness symmetry in individuals with stroke using ultrasound [18,21]. These studies [18,21] showed that TrA thickness at rest is almost identical between the two sides of the body in both hemiparetic individuals and controls. In contrast, the contraction thickness ratio during the ADIM was higher in the non-paretic side than in the paretic side of individuals who had experienced a stroke [18]. While some studies reported that lateral trunk flexion strength was lower on the affected side compared with the unaffected side [22], others reported the opposite [23]. This indicates that further examination of TrA thickness asymmetry after stroke is worthwhile.

Therapeutic exercise programs that aim to improve trunk stability are considered essential to increasing the functional ability and quality of life in individuals who have had a stroke [9]. The identification of muscle atrophy as well as impairments in performing core stability exercises, such as the ADIM, using ultrasound may improve the design and effectiveness of exercise interventions in these patients. The purpose of this study was to examine, firstly, the differences in the thickness and contraction of the TrA between individuals with stroke and matched controls, and, secondly, to report on side asymmetries in the TrA thickness at rest and exercise in both groups.

## 2. Materials and Methods

### 2.1. Participants

Patients who were institutionalized in a local rehabilitation center and age-matched individuals who served as controls were invited to participate in this project. To be included in the stroke group, the following inclusion criteria had to be met: a) Presence of hemispheric lesion based on clinical examination and diagnostic imaging techniques; b) stoke occurred for a period of 1 to 6 months prior to testing; c) patients had hemiparesis or hemiplegia after stroke; d) no cognitive or mental deficits or difficulties in understanding and following the instructions were present, as measured using Mini-Mental State Examination (MMSE) results; e) no previous musculoskeletal conditions or any conditions that influence trunk stabilization or lead to acute or chronic low back pain; and f) they had a score greater than 61 in the Barthel index scale, including mild bladder control problems (score 1 in the same scale) and independent walking with the use of any aid (score 2 in the same scale). Patients were excluded if they had low back pain, were unable to provide and sign an informed consent, had a history of abdominal or trunk surgery, or had other neurological disorders or impairments.

Nine patients met the inclusion criteria and participated in the project (age 71.87 ± 8.42 years, body mass 65.75 ± 6.54 kg, height 162.12 ± 3.75 cm; BMI 24.84 ± 1.93, Barthlet index score 84 ± 5.11). Subsequently, 10 individuals who were matched to the patient group based on age and body mass index (BMI) (age 71.27 ± 6.53 years, body mass 65.81 ± 5.05 kg, height 162.81 ± 4.06 cm, BMI = 25.37 ± 2.07, Barthlet index score 98.31 ± 2.13), were recruited from a local residential care unit for the elderly. To be included in this group, individuals did not meet any of the inclusion and exclusion criteria as the stroke patients. Participation in the study was voluntary and patients fully understood the purpose of the study. All participants were informed about the tests and the use of the results and were asked to sign a written informed consent statement. The study was approved by university review boards (ERC-007/2020 and 18615520) and followed the principles outlined in the Declaration of Helsinki.

### 2.2. Experimental Design

All subjects were provided oral explanations and procedural instructions regarding the purpose of the experiment. Individuals were examined in two sessions. The first session aimed to familiarize the participants with the protocol. This included demonstration of the abdominal drawing-in maneuver (ADIM), the bridge exercise, and the bridge exercise in combination with the ADIM. Individuals then performed no less than 6–8 repetitions in each condition, under the guidance of the experimenters. US measurements were also collected to establish reliability. During the second session, which took place within 4–7 days after the first one, muscle thickness measurements were performed.

### 2.3. Instrumentation

Ultrasound (US) images of ΤRA were obtained using a portable brightness mode (B-mode) US imaging device (GE Logiq e, V2, General Electric, Milwaukee, WI, USA) and a multifrequency linear-array probe (L-RS, 5–13 MHz, 40.0 mm field-of-view, General Electric, Milwaukee, WI, USA). GE e Logic LogicView software was used to produce and digitally store images of each area in real time.

### 2.4. Procedures

TrA thickness was measured in each of the following four conditions: At rest mode (Rest), the ADIM, bridge, and bridge in combination with ADIM (bridge-ADIM). In the resting position, the participants were instructed to hold a hook-lying position (supine position with knees bent at 60° and feet on the bed), placing their arms crossed on the chest. They were asked to breathe normally, avoiding unnecessary movement of the body and muscle contraction. They were then instructed to perform the ADIM by taking one last breath and simultaneously exhale and gently pull their lower abdomen inwards. The verbal guidance was “feel your navel moving towards the spine while keeping the abdominal muscles relaxed”. Participants kept this position for approximately 5 s. Following a rest period of 5 min, the bridge exercise was performed. From the hook-lying position, the participants moved their arms next to the body and performed a pelvic lift to the point where thee shoulders, hips, and knees formed a straight line. After another resting period, the bridge-ADIM efforts were performed by first assuming the bridge position and then performing the ADIM for a period of 5 s.

**Figure 1:**
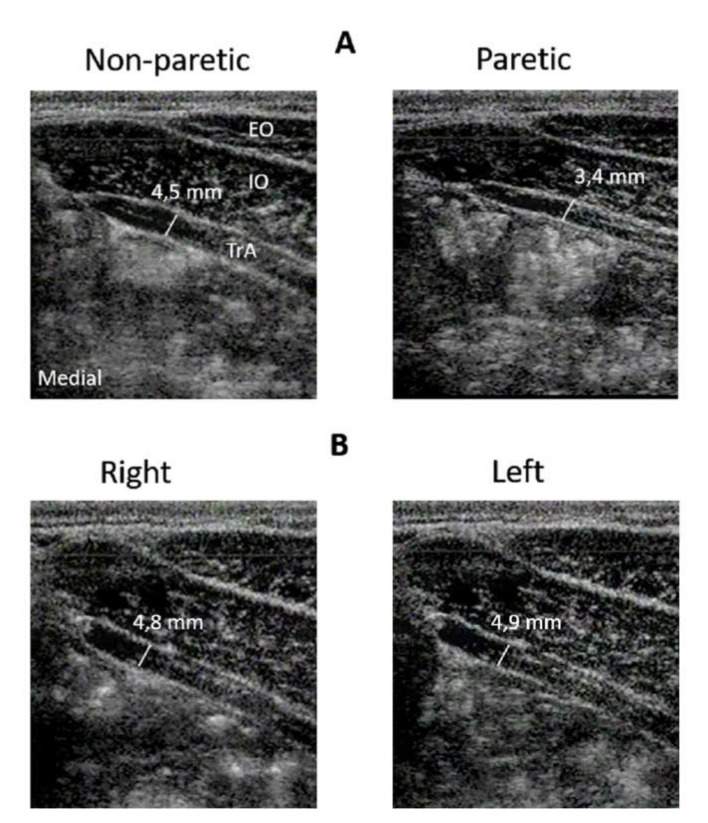
Example ultrasound images of the transversus abdominal (TrA) muscle from A. an individual with hemiparesis and B. control group from each side of the body at rest. The TrA muscle is located in this image as a layer under the external (EO) and internal (IO) oblique abdominals. Thickness measurements were made between the superficial and deep borders of the TrA muscle.

Measurements were taken from the TrA from the participants’ left and right sides. A minimum of 3 scans were taken per side, per condition (Figure 1). Ultrasound gel was applied liberally to the areas of imaging to ensure good sonic coupling between the transducer and the skin.

To assess the TrA thickness, the tester located the linear probe at the middle of the 11th costal cartilage and iliac crest, perpendicularly to the midaxillary line, as proposed by Teyhen et al. (2007). To standardize the position of the TrA on the ultrasound screen, the anterior edge of the TrA was visualized on the far left of the screen. The image was then frozen and the muscle thickness was calculated as the perpendicular distance between the upper and lower fascia, approximately 2.5 cm from the left edge of the screen.

The dependent variables were the TrA thickness measured with the participants at rest, during ADIM, bridge, and bridge-ADIM. In addition, for each exercise condition, the contraction thickness ratio (CTR), averaged for both sides, was calculated as the percentage change in the muscle thickness from rest to contraction. The difference in thickness between the left and the right sides was first estimated and then the absolute value (irrespective of the direction of the differences) of the percentage difference in thickness between the two sides (“symmetry side index”) was further analyzed.

### 2.5. Statistical Analyses

Intraclass correlation coefficients (ICCs) were calculated for the assessment of the intra-examiner reliability between the first and second session measurements [24]. An ICC value ≤0.50 was considered low, 0.50 to 0.75 moderate, ≥0.75 good, and ≥0.90 excellent. To assess the measurement precision, the standard error of measurement (SEM) was calculated by using the following equation: SEM = SD × √ (1 − ICC).

Data were checked for normality using the Kolmogorov–Smirnov test. A two-way analysis of variance (ANOVA) was used to determine the differences in the TrA thickness between body sides and testing conditions. Group differences in the symmetry index and CTR were checked using separate ANOVA designs. Post hoc Tukey tests were performed in order to investigate possible differences between exercises, for each muscle side separately. The level of statistical significance was set to a = 0.05.

## 3. Results

### 3.1. Reliability

The reliability results are presented in Table 1. The ICC_2,1_ ranged from 0.76 to 0.99 and the SEM ranged from 0.01 to 0.23 cm in the patient group. The corresponding values for the control group were from 0.82 to 0.98 and from 0.01 to 0.16 cm for the ICC_2,1_ and SEM, respectively.

### 3.2. Muscle Thickness

The muscle thickness group values are presented in Table 2. The ANOVA showed a statistically significant group by side interaction effect on muscle thickness (*F*_1.17_ = 8.50, *p* < 0.05). Post hoc analysis showed that in each testing condition, muscle thickness was significantly lower in the stroke than the control group (*p* < 0.05). Further, the thickness was significantly lower at rest compared with all exercise conditions (*p* < 0.05). However, no differences in thickness between exercises were found (*p* > 0.05).

### 3.3. Contraction Thickness Ratio

**Figure 2:**
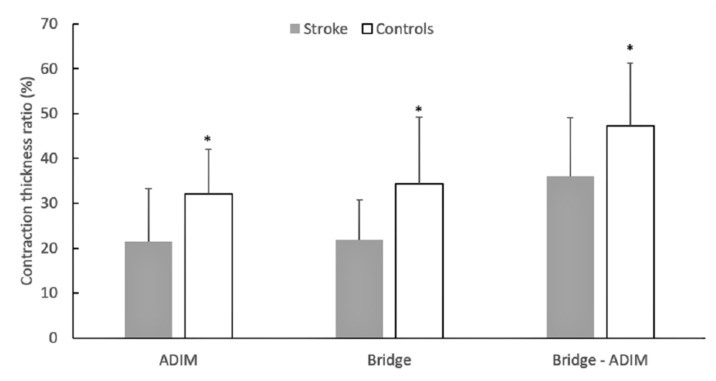
Mean group values of the contraction thickness ratio in poststroke hemiparetic patients and controls at rest, the abdominal drawing maneuver (ADIM), the bridge, and the bridge-ADIM condition. Error bars indicated standard deviation; * significant group difference at *p* < 0.05.

The CTR index values ranged from 21.42 ± 12.29% to 36.02 ± 14.51% in patients and from 32.05 ± 7.10% to 47.33 ± 13.42% in controls (Figure 2). The ANOVA showed a significant main effect for group (*F*_1.17_ = 6.71, *p* < 0.05) as stroke patients had significantly lower CTR values than controls, in all testing conditions. In addition, CTR was greater in the bridge-ADIM condition compared to either the ADIM or bridge exercises (*p* < 0.05).

### 3.4. Symmetry Index

**Figure 3:**
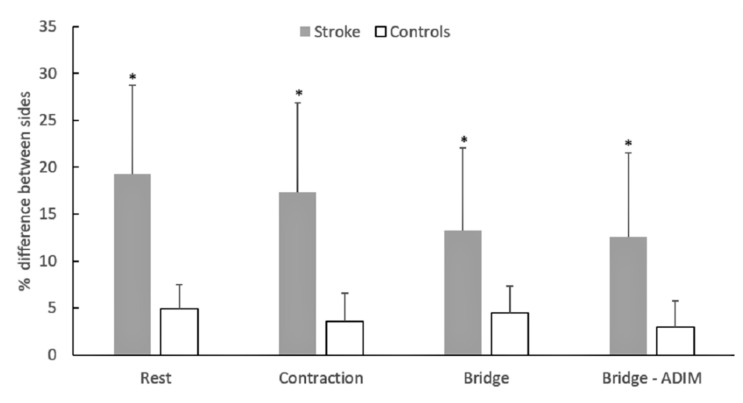
Mean group values of the absolute symmetry thickness between body sides in poststroke hemiparetic patients and controls at rest, the abdominal drawing maneuver (ADIM), the bridge, and the bridge-ADIM condition. Error bars indicated standard deviation; * significant group difference at *p* < 0.05.

The percentage (absolute) symmetry index values ranged from 12.59 ± 6.43% to 19.31 ± 10.43% in patients and from 3.01 ± 2.47% to 4.47 ± 2.87% in controls (Figure 3). The ANOVA showed a significant main effect for group (*F*_1.17_ = 20.19, *p* < 0.05) as stroke patients had significantly greater symmetry index values than controls, in all testing conditions.

## 4. Discussion

The main findings of this study are that individuals with poststroke hemiparesis display a lower TrA thickness at rest and CTR compared with controls. TrA thickness was greater in the non-paretic side than the paretic side at rest. The absolute value of the symmetry in the thickness between the two body sides was greater in patients than controls.

The lower TrA thickness at rest in the patient group (Table 2) is indicative of atrophy of this deep abdominal muscle of both sides. To our knowledge, only a few studies have examined TrA thickness in individuals with stroke using ultrasound. Specifically, our results are in line with those reported by Kim et al. [18], who found an average difference of 25 mm (~45%) in the resting TrA thickness in individuals with hemiparesis and controls. This is in line with a review study that showed that muscle (sarcopenia) loss is a secondary but important symptom that occurs after stroke [3]. There are various factors that may have contributed to this finding. First, in this study, the two groups were matched for age and BMI to control for the potential influence of these factors in group comparisons. Second, poststroke patients, especially those who are institutionalized, have very low physical activity levels for a long period after stroke [25,26]. Further, it has been suggested that cerebral damage may induce an increase in the rate of motor unit denervation, synaptic reorganization, and local neuronal (vegetative) imbalances [3], which cumulatively may lead to selective sarcopenia.

Another finding of this study was the observation that patients had difficulty in contracting the TrA during exercises in comparison to controls (Figure 2). This is in line with the study by Kim et al. [18], who reported that the increase in thickness during the ADIM was lower in patients than controls. Our findings extend these observations as we noticed similar group differences not only during ADIM but also in another frequent core stability exercise position (bridge) as well as the combination of bridge with ADIM (Figure 3). While differences in the resting muscle thickness may be indicative of atrophy of the TrA, a lower CTR represents a reduced ability to contract the TrA during exercise. There is evidence that activation of the postural trunk muscles is altered in poststroke patients [27,28], such as a delayed onset of muscle activation [27] and reduced anticipatory activity [6]. If stroke results in additional motor unit denervation [3], then this would ultimately cause altered activation of the trunk muscles. In addition, the presence of greater intramuscular fat in patients [29] might have resulted in lower recruitment capacity of the trunk muscles [30]. Collectively, within the limitations of the cross-sectional design of the present study, it appears that such patients not only have atrophy of the deep abdominal muscles, but they also have difficulties in contracting this muscle during a typical stabilization exercise. It is clear, however, that more information is necessary to verify the above findings.

In the present study, the patients showed an approximately 20% greater TrA thickness difference between sides than controls, in the rest and exercise conditions (Figure 3). A meta-analysis showed that individuals at least 6 months poststroke have significantly less muscle mass in their paretic limbs compared with their nonparetic limbs, which may range between 4.5% and 14.5% [29]. However, very few studies have examined the asymmetry in the muscle mass or thickness between the left and right side of the trunk [9,18]. Our results are in contrast to a previous study [18], which reported no differences in TrA thickness at rest between patients and controls. However, Tsuji et al. [9] reported that 40% of patients with hemiparesis had greater paravertebral muscle cross sectional area on the contralateral side while in another 40% of the patients, no bilateral differences were observed. It was suggested that patients with greater paravertebral muscle CSA on the contralateral side tended to have more severe functional limitations [9]. This may explain the difference between our findings and those reported by Kim et al. [18], who examined non-institutionalized and much younger (52 years) individuals compared to the ones examined in the present study. It is clear, however, that more research is required to examine trunk muscle size asymmetries between sides in individuals with stroke in relation to functional limitations.

One may suggest that selective atrophy of the paretic side could also be a result of lower TrA recruitment during the daily tasks of these patients. However, our results did not show differences in the TrA thickness between sides in each exercise condition (Table 2). To further explain these results, we noticed that while five patients had greater TrA thickness during exercise in one side, there were three patients who showed exactly the opposite. Hence, when the direction of the differences between sides was not taken into consideration, the patient group displayed a much greater index of asymmetry in the thickness between sides (Figure 3). Research studies have shown less anticipatory electromyographic (EMG) responses of the latissimus dorsi and erector spinae and rectus abdominis to paretic arm abduction on the paretic side in poststroke hemiparetic patients [27]. However, these results refer to responses to unilateral movements, and, hence, a direct comparison with our findings is difficult. Both our study and the study by Kim et al. [18] found that patients had a greater TrA contraction thickness than controls in the non-paretic compared to the paretic side. In contrast, Bae et al. [31] found greater EMG activation of the internal oblique and the external oblique abdominals in the paretic than the non-paretic side during standard bridge exercise and during a bridge exercise combined with unilateral arm movements. The reasons for these conflicting findings are unclear. It has been suggested that the observed greater trunk muscle activation in the paretic side reflects a greater influence of stroke on the functional use on this side even though unilateral stroke at the level of the central nervous system affects both sides of the trunk [6,32,33]. It is likely that the reduced physical activity levels and a lower walking independency have led some patients to perform these exercises by relying mainly on the contraction of their non-paretic TrA muscle relative to the paretic one, while others have developed exactly the opposite strategy. However, the possibility that patients have recruited other synergist muscles, such as the erector spinae, oblique abdominals, and lumbar multifidus, in a different way than the TrA cannot be ignored.

The results may have some implications for exercise interventions for patients with hemiparetic stroke. Exercise programs that aim to improve trunk stability are particularly beneficial for improving the functional capacity of patients with stroke [9,34]. There is evidence that the anticipatory muscle activation strategy to arm movement is characterized by early TrA pre-activation in individuals without pathologies [14]. Further, there is evidence that the ability to activate the deep trunk musculature depends on proprioception, motor learning ability, and respiratory patterns [35]. Consequently, exercises that assist in the recruitment of this muscle, such as the ones applied in the present study, may be beneficial for improving muscle atrophy and trunk stabilization after stroke. Our experimental design does not allow conclusions regarding the effects of bridging exercises that involve one side of the body. We can only speculate that the improvement of bilateral activation asymmetries may take place after patients have improved their general capacity to perform such exercises. Further research is necessary to verify this suggestion.

This study has several limitations. First, the number of participants, especially in the patient group, was small, but this is mainly due to the selection criteria, which excluded patients with heavy ambulatory or mental problems, which are very frequent following stroke. Second, although individuals who satisfied the inclusion criteria had a similar profile, the history of physical rehabilitation prior to measurement is unknown. It is highly possible that previous exercise or therapeutic intervention influenced the results of this study. Third, we quantified TrA thickness using ultrasound-based software, rather than using a separate imaging analysis software. This was for the benefit of the participants as the examination time was reduced. Finally, measurements were performed by the same examiner, which affects their generalizability to other examiners. Since the procedures followed in the present study were carefully applied based on previously applied protocols, it is very likely that trained examiners with similar experience would produce similar results.

## 5. Conclusions

The reduced TrA thickness displayed by patients with hemiparesis in this study is indicative of muscle atrophy in both sides of the trunk. The lower CTR displayed by patients compared to controls indicates that patients cannot fully activate their muscles during a typical core stability exercise. Consequently, core stability programs may be beneficial for improving core stability, especially of the paretic side, after poststroke hemiparesis.

## Figures and Tables

**Table 1:** Reliability values for the transversus abdominis thickness values for both groups.

Effect	Stroke				Controls			
Side	Left		Right		Left		Right	
Condition	ICC	SEM	ICC	SEM	ICC	SEM	ICC	SEM
Rest	0.99	0.01	0.99	0.01	0.97	0.01	0.98	0.01
ADIM	0.83	0.24	0.83	0.15	0.82	0.16	0.94	0.09
Bridge	0.78	0.18	0.77	0.17	0.92	0.11	0.93	0.12
Bridge-ADIM	0.77	0.26	0.76	0.23	0.86	0.13	0.90	0.14

ADIM = Abdominal drawing-in maneuver, Bridge = pelvic lift, Bridge-ADIM = Bridge with ADIM; ICC: Intraclass Correlation Coefficient, SEM = standard error of measurement.

**Table 2:** Mean (± SD) thickness of the transversus abdominis (mm) in each testing condition.

Effect	Controls		Stroke	
Condition	Right	Left	Right	Left
Rest	3.74 ± 0.58	3.73 ± 0.54	3.18 ± 0.92	2.82 ± 0.99
ADIM	4.92 ± 0.67	4.91 ± 0.71	3.85 ± 0.93	3.81 ± 1.64
Bridge	5.02 ± 0.62	4.96 ± 0.55	3.73 ± 1.28	3.55 ± 1.42
Bridge-ADIM	5.44 ± 0.53	5.46 ± 0.57	4.48 ± 1.13	4.01 ± 1.91

ADIM = Abdominal drawing-in maneuver, Bridge = pelvic lift, Bridge-ADIM = Bridge with ADIM.
